# Pyrolysis of Denim Jeans Waste: Pyrolytic Product Modification by the Addition of Sodium Carbonate

**DOI:** 10.3390/polym14225035

**Published:** 2022-11-21

**Authors:** Junghee Joo, Heeyoung Choi, Kun-Yi Andrew Lin, Jechan Lee

**Affiliations:** 1 Department of Global Smart City, Sungkyunkwan University, Suwon 16419, Republic of Korea; 2 Department of Environmental Engineering & Innovation and Development Center of Sustainable Agriculture, National Chung Hsing University, Taichung 40227, Taiwan; 3 School of Civil, Architectural Engineering, and Landscape Architecture, Sungkyunkwan University, Suwon 16419, Republic of Korea

**Keywords:** thermochemical conversion process, waste recycling, waste-to-energy, waste treatment, synthetic fiber

## Abstract

Quickly changing fashion trends generate tremendous amounts of textile waste globally. The inhomogeneity and complicated nature of textile waste make its recycling challenging. Hence, it is urgent to develop a feasible method to extract value from textile waste. Pyrolysis is an effective waste-to-energy option to processing waste feedstocks having an inhomogeneous and complicated nature. Herein, pyrolysis of denim jeans waste (DJW; a textile waste surrogate) was performed in a continuous flow pyrolyser. The effects of adding sodium carbonate (Na_2_CO_3_; feedstock/Na_2_CO_3_ = 10, weight basis) to the DJW pyrolysis on the yield and composition of pyrolysates were explored. For the DJW pyrolysis, using Na_2_CO_3_ as an additive increased the yields of gas and solid phase pyrolysates and decreased the yield of liquid phase pyrolysate. The highest yield of the gas phase pyrolysate was 34.1 wt% at 800 °C in the presence of Na_2_CO_3_. The addition of Na_2_CO_3_ could increase the contents of combustible gases such as H_2_ and CO in the gas phase pyrolysate in comparison with the DJW pyrolysis without Na_2_CO_3_. The maximum yield of the liquid phase pyrolysate obtained with Na_2_CO_3_ was 62.5 wt% at 400 °C. The composition of the liquid phase pyrolysate indicated that the Na_2_CO_3_ additive decreased the contents of organic acids, which potentially improve its fuel property by reducing acid value. The results indicated that Na_2_CO_3_ can be a potential additive to pyrolysis to enhance energy recovery from DJW.

## 1. Introduction

Textiles in municipal solid waste is mainly discarded clothing with other sources such as carpets, footwear, sheets, and towels. According to United States Environmental Protection Agency (U.S. EPA), approximately 17 million tons of textile waste ended up in landfills in 2018 [1]. The U.S. EPA also estimated that nearly 5% of all landfill space is occupied by textile waste. Landfilling textile waste causes environmental issues such as the formation of greenhouse gases upon decomposition and the contamination of groundwater [2]. Some kinds of textiles (e.g., synthetic ones) require longer than 200 years to decompose in landfills [3]. In addition to the generation of tremendous amounts of textile waste, synthetic textile fibers are manufactured using fossil fuel resources (e.g., natural gas and crude oil) as the feedstock. The production, consumption, and postindustrial waste handling of synthetic textile fibers not only generate greenhouse gas emissions, but also release microplastics [4]. Therefore, the strategy for disposal of textile waste has changed from landfill-based solution to recycling-based solution.

Upcycling is a kind of recycling, which converts lower-value substances to higher-value products [5]. Pyrolysis has gained increasing attention as a feasible waste upcycling process because it is an effective process for the treatment of waste materials in a heterogeneous complex nature [6]. Recently, pyrolysis has been widely studied to not only recover energy from waste substances [7] but also transform waste materials into value-added products such as hydrogen gas [8] and commodity chemicals [9]. For instance, a pyrolysis process using a cobalt-based catalyst has been suggested to transform textile waste into combustible products, as a method of waste-to-energy [10,11]. Kim et al. has recently reported that calcium carbonate-based catalysts enhanced nylon monomer recovery from fishing net waste made of nylon [12]. In particular, sodium carbonate (Na_2_CO_3_) has been used to modify the characteristics of pyrolytic products produced from different carbonaceous feedstocks such as coal [13], pine sawdust [14], and Crofton weed [15,16]. In this respect, pyrolysis in the presence of Na_2_CO_3_ can be a promising option for the upcycling of textile waste by modifying the pyrolytic products characteristics (e.g., the enhancement of the production of combustible gases and improvement of fuel properties of pyrolytic liquid).

There has been no study on Na_2_CO_3_-mediated pyrolysis of textile waste so far. This is the first study to explore the impacts of Na_2_CO_3_ on the pyrolysis of textile waste containing both natural and synthetic fibers such as denim jeans waste (DJW). The DJW was chosen as a textile waste surrogate as the generation of DJW has continuously increased due to the continuous growth of global denim jeans market size [17]. The present study was aimed at supporting the creation of effective energy recovery from textile waste based on the pyrolysis in the presence of Na_2_CO_3_. It was also expected that this study proposes a potential method to feasibly recover energy from textile waste.

## 2. Materials and Methods

### 2.1. Feedstock

DJW was collected at clothing donation centers located in Suwon, Gyeonggi Province, Republic of Korea. After collection, impurities in DJW were thoroughly removed using an air blower followed by cutting to slips with a thickness of 2 cm (Figure S1). Na_2_CO_3_ (purity: ≥99.5%) was purchased, provided from Sigma-Aldrich brand in Merck (Burlington, MA, USA).

Ultimate analysis of DJW was performed using an elemental analyzer (model: 628 series; LECO, St. Joseph, MI, USA). Proximate analysis of DJW (dry basis) was conducted according to ASTM standard methods (D3175 for volatile matter and D3174 for ash). The difference between the initial DJW sample mass and the sum of volatile matter and ash was considered the content of fixed carbon.

### 2.2. Pyrolysis Experiment and Product Analysis

The pyrolysis experiments were conducted at 400–800 °C in a continuous flow pyrolyser (described in detail in Figure S2). A tubular reactor (outside diameter: 25 mm; inside diameter: 21 mm; length: 0.6 m) made of quartz was heated by a split-hinge tube furnace (Thermo Scientific, Waltham, MA, USA). For a pyrolysis experiment, 1 g of feedstock (i.e., DJW) was loaded in the center of the quartz tube and fixed with quartz wool on both sides of the feedstock. For the pyrolysis experiment conducted in the presence of Na_2_CO_3_, 10 wt% Na_2_CO_3_ was added to the feedstock as the Na_2_CO_3_ loadings below 10 wt% did not have distinct effects on the production of pyrolysates for the DJW pyrolysis.

Nitrogen gas (ultra-high purity) was continuously supplied into the tubular reaction at a flow rate of 100 mL min^−1^, which was controlled by a mass flow controller (Brooks Instrument, Hatfield, PA, USA). The product stream passes through a condenser composed of an ice trap (−1 °C) and four dry ice/acetone traps (−50 °C each) connected in a series.

The condensable fraction of the product stream was collected in the condenser. The collected condensable samples were analyzed using a gas chromatograph equipped with a mass spectrometer (GC/MS; Agilent Technologies, Santa Clara, CA, USA). The non-condensable fraction of the product stream (i.e., the fraction that was not collected in the condenser) further passed through a micro gas chromatograph (micro GC; INFICON, Bad Ragaz, Switzerland) to analyze non-condensable gases evolved from the pyrolysis of DJW. Detailed conditions used for the micro GC and GC/MS analyses are given in Tables S1 and S2.

## 3. Results and Discussion

### 3.1. Feedstock Characterization

Table 1 summarizes the results of ultimate and proximate analyses of DJW on dry basis. The DJW feedstock was mostly composed of carbon (45 wt%) and oxygen (32.16 wt%). Considerable amounts of hydrogen (6.35 wt%) and ash (15.09 wt%) were also found. The contents of nitrogen and sulfur were negligible. The proximate analysis of DJW confirmed that the DJW feedstock mostly consisted of volatile matter (84.1 wt%).

### 3.2. Pyrolysis of Denim Jeans Waste

The pyrolysis of DJW resulted in pyrolysates in three different phases, such as pyrolytic gas, pyrolytic oil, and solid residue. In the primary stage of DJW pyrolysis, pyrolytic volatiles were released from the feedstock, and then they were thermally degraded to condensable compounds (i.e., liquid phase pyrolysate) [18]. The condensable compounds further underwent thermal decomposition resulting in lighter molecules, such as non-condensable gases (i.e., gas phase pyrolysate), in the secondary stage of DJW pyrolysis [18]. Solid phase pyrolysate was the residual solid after all pyrolytic volatiles were released from DJW [19].

**Figure 1:**
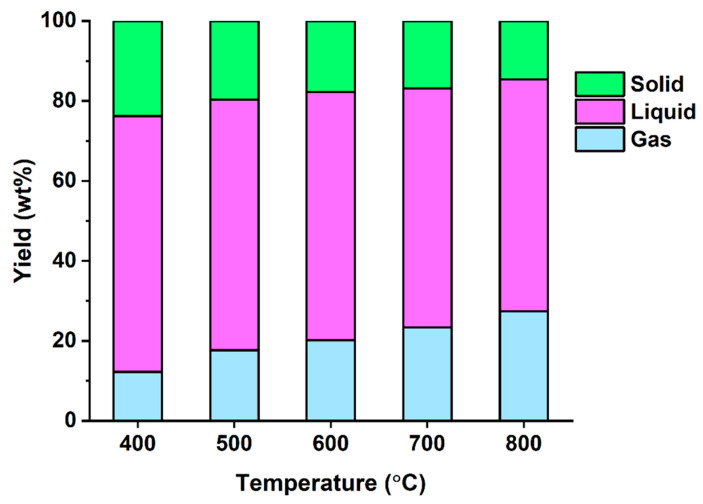
Mass balance of pyrolysate made from DJW without Na_2_CO_3_ as a function of pyrolysis temperature. Average values of triplicate are reported with and standard deviations of the average values of 3–4%.

Figure 1 shows that the yields of three phase pyrolysates produced from DJW without Na_2_CO_3_ at varied temperatures. A clear trend was observed: an increase in pyrolysis temperature increased the yield of gas phase pyrolysate and decreased the yields of liquid phase pyrolysate and solid residue. For instance, the yield of gas phase pyrolysate increased from 12.2 wt% to 27.4 wt% with increasing the temperature from 400 °C to 800 °C, while the total yield of liquid and solid phase pyrolysates decreased from 87.8 wt% to 72.6 wt%. This clearly indicates that the release of pyrolytic volatiles and thermal degradation of the pyrolytic volatiles were promoted by increasing pyrolysis temperature in the pyrolysis of DJW.

**Figure 2:**
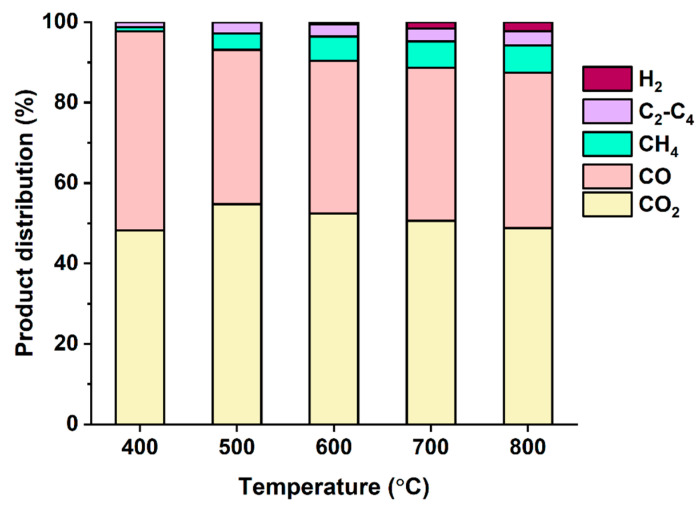
Product distribution of the gas phase pyrolysate made from DJW without Na_2_CO_3_ as a function of pyrolysis temperature. Average values of triplicate are reported with and standard deviations of the average values of 3–4%.

The gas phase pyrolysate was composed of combustible gases, such as carbon monoxide (CO), hydrogen (H_2_), and C_1_–C_4_ hydrocarbons, and carbon dioxide (CO_2_), as presented in Figure 2. Major components of the gas phase pyrolysate was CO and CO_2_ at all temperatures tested. An increase in pyrolysis temperature increased the selectivities toward H_2_ and methane (CH_4_) in return for the selectivity toward CO_2_. For example, when increasing the pyrolysis temperature from 600 °C to 800 °C, the H_2_ and CH_4_ selectivity increased from 6.6% to 9.2%, while the CO_2_ selectivity decreased from 52.4% to 48.8%. This was most likely attributed to the dehydrogenation and methanation reactions expedited at higher temperatures [20].

The DJW-derived liquid phase pyrolysate was a mixture of a wide variety of chemical compounds that could be classified as acids, alcohols, aldehydes, ketones, esters, furans, dioxolanes, hydrocarbons, sugars, and other miscellaneous compounds, as summarized in Table 2. According to the fiber content provided in clothing labels, the average composition of the DJW feedstock was cotton (~90%), polyester (~8%), and elastane (1–2%). Acids, alcohols, aldehydes, ketones, furans, and sugars should originate from natural fiber such as cotton. Esters, dioxolanes, and hydrocarbons were most likely derived from synthetic fibers such as polyester and elastane via thermal cracking of polymeric bonds. N-containing species were associated with blue dye used for manufacturing denim fabric. As found in Table 2, most of the compounds were acids, alcohols, ketones, and sugars. The change in pyrolysis temperature did not markedly affect the product distribution of the liquid phase pyrolysate.

### 3.3. Effects of Na_2_CO_3_ Addition to Pyrolysis of Denim Jeans Waste

**Figure 3:**
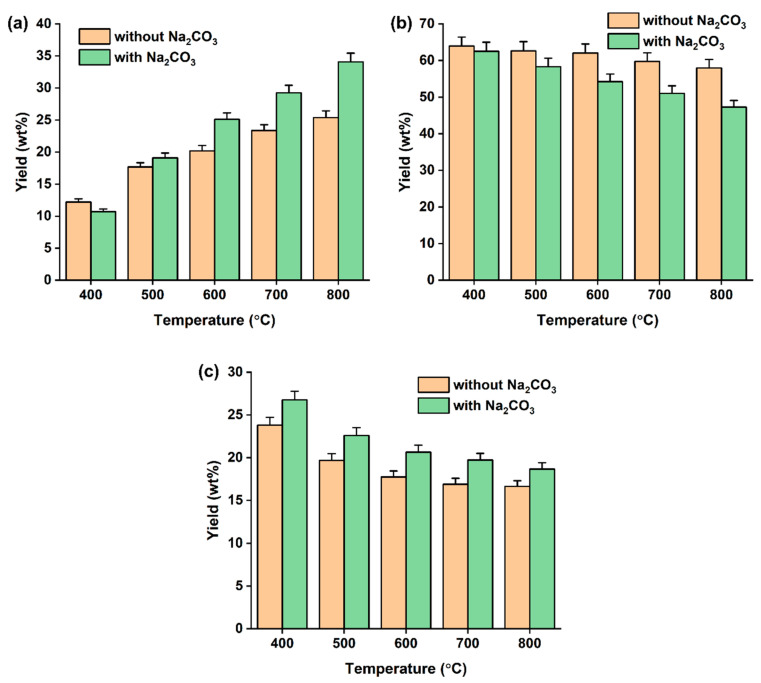
The yield of pyrolysates made from DJW with and without Na_2_CO_3_ at varied pyrolysis temperatures: (**a**) gas phase pyrolysate, (**b**) liquid phase pyrolysate, and (**c**) solid phase pyrolysate. Average values of triplicate are reported with standard deviations given as error bars.

In Figure 3, the yields of three phase pyrolysates obtained with and without Na_2_CO_3_ are compared. The addition of Na_2_CO_3_ to the DJW pyrolysis increased the yields of gas and solid phase pyrolysates while it decreased the yield of liquid phase pyrolysate at all tested temperatures. For instance, the gas phase pyrolysate yield achieved with Na_2_CO_3_ was 34% higher than that achieved without Na_2_CO_3_ at 800 °C. As aforementioned, the solid phase pyrolysate yield is highly associated with the release of pyrolytic volatiles from DJW, and the liquid phase pyrolysate yield is highly associated with thermal degradation of the pyrolytic volatiles. Therefore, the increase in the yields of gas and solid pyrolysates and the decrease in the yield of liquid pyrolysate were most likely because Na_2_CO_3_ enhanced the thermal degradation of the pyrolytic volatiles released from DJW during the pyrolysis.

**Figure 4:**
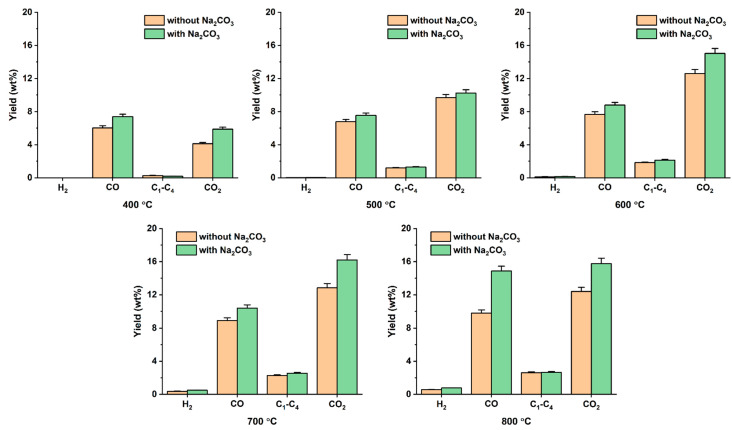
Non-condensable gas yields obtained by the pyrolysis of DJW with and without Na_2_CO_3_ at varied pyrolysis temperatures. Average values of triplicate are reported with standard deviations given as error bars.

The addition of Na_2_CO_3_ had considerable effects on the composition of the gas phase pyrolysate. In Figure 4, the yields of non-condensable gases contained in the gas phase pyrolysates obtained with and without the addition of Na_2_CO_3_ are compared. It was clearly observed that the addition of Na_2_CO_3_ enhanced the yields of H_2_, CO, and CO_2_ at 400–800 °C. H_2_ is formed through secondary decomposition and recombination of C–H groups and aromatic C=C bonds [21]. Alkaline sodium species in Na_2_CO_3_ (e.g., Na^+^) should contribute to the formation of H_2_ [22]. The addition of Na_2_CO_3_ most likely promoted decarboxylation and decarbonylation, resulting in higher CO_2_ and CO yields, respectively [23]. Furthermore, in the presence of Na_2_CO_3_, reactions between Na^+^ and –COH and –COOH groups leads to phenolic sodium (–CONa) and carboxylate sodium (–COONa), respectively, thereby releasing H^+^ (Equations (1) and (2)) [24]. The –CONa and –COONa species can react with solid phase carbon, resulting in CO (Equations (3) and (4)) [24]. The extent of the enhancement of H_2_, CO, and CO_2_ tended to be greater as the pyrolysis temperature increased, meaning that the above-mentioned reactions were more promoted at higher temperatures. The yield of C_1_–C_4_ hydrocarbons was not markedly changed by adding Na_2_CO_3_ to the DJW pyrolysis. The higher heating value of the gas phase pyrolysate obtained with Na_2_CO_3_ was up to 28% higher than that without Na_2_CO_3_, ranging from 8 MJ kg^−1^ to 12 MJ kg^−1^ (calculated based on the heat of combustion of individual combustible gas). In other words, the addition of Na_2_CO_3_ to the DJW pyrolysis can help to improve the energy content of the gas phase pyrolysate derived from the DJW feedstock.

−COH + Na^+^ → −CONa + H^+^ (1)

−COOH + Na^+^ → −COONa + H^+^ (2)

−CONa + C(s) → −CNa + CO(g)(3)

−COONa + C(s) → −CONa + CO(g)(4)

**Figure 5:**
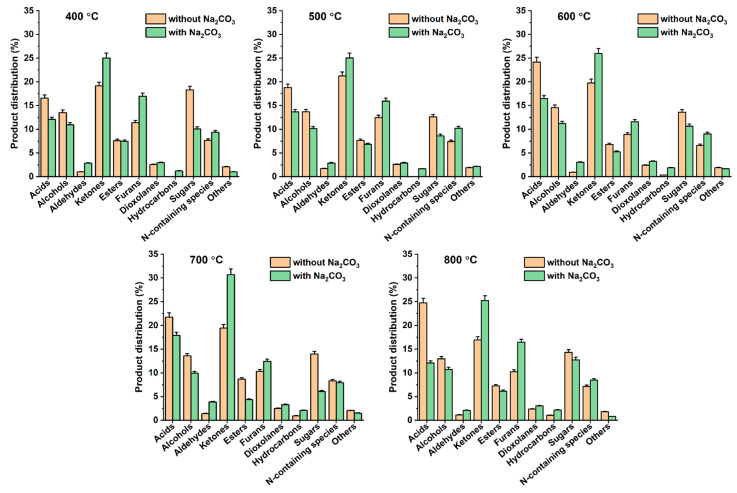
Product distributions of the liquid phase pyrolysates of DJW with and without Na_2_CO_3_ at varied pyrolysis temperatures. Average values of triplicate are reported with standard deviations given as error bars.

Figure 5 showed the comparisons of the liquid phase pyrolysates made from DJW with and without Na_2_CO_3_. The addition of Na_2_CO_3_ decreased the contents of acids, alcohols, esters, and sugars, while it increased the contents of ketones, furans, hydrocarbons, and N-containing species at all the tested pyrolysis temperatures. Among the compounds in the liquid phase pyrolysates, acids can deteriorate the fuel property of pyrolytic liquid because acid species increase the acidity of the pyrolytic liquid; thus, it can cause corrosion issues in engines and boilers [25]. The product distribution of acids in the liquid phase pyrolysate produced without Na_2_CO_3_ ranged from 17% to 25%; however, the product distribution of acids in the liquid phase pyrolysate produced with Na_2_CO_3_ ranged from 12% to 17%. This evidently indicates that the addition of Na_2_CO_3_ had a deoxidation effect on the liquid phase pyrolysate derived from DJW. As a result, the pyrolysis of DJW in the presence of Na_2_CO_3_ could improve the fuel property of pyrolytic liquid by decreasing its acid value.

## 4. Conclusions

Here in this study, the pyrolysis of DJW was carried out in a continuous flow pyrolyser with and without the addition of Na_2_CO_3_ as a method for the upcycling of textile waste. The increase in pyrolysis temperature increased the yield of gas phase pyrolysate and decreased the yields of liquid and solid phase pyrolysates most likely due to the enhanced thermal cracking of pyrolytic volatiles released during the DJW pyrolysis. The addition of Na_2_CO_3_ further enhanced the yield of the gas phase pyrolysate reaching 34.1 wt%. The addition of Na_2_CO_3_ increased the yield of combustible gases (e.g., H_2_ and CO) compared with the DJW pyrolysis without Na_2_CO_3_, thereby increasing the energy content of the gas phase pyrolysate. The major components of the liquid phase pyrolysate were acids, alcohols, aldehydes, ketones, furans, and sugars. In the presence of Na_2_CO_3_, the content of organic acids was decreased, which potentially improves the fuel property of the pyrolytic liquid by decreasing its acid value.

## Figures and Tables

**Table 1:** Results of proximate and ultimate analyses of DJW (dry basis).

Analysis	Composition	Content (wt%)
Ultimate analysis	C	45.00
	H	6.35
	O	32.16
	N	0.20
	S	1.20
	Ash	15.09
	Total	100
Proximate analysis	Volatile matter	84.10
	Fixed carbon	0.81
	Ash	15.09
	Total	100

**Table 2:** Product distribution of the liquid phase pyrolysate made from DJW without Na_2_CO_3_ at varied pyrolysis temperatures (unit: GC/MS area%).

Component	400 °C	500 °C	600 °C	700 °C	800 °C
Acids	19.6	18.8	19.2	18.8	19.7
Alcohols	13.5	13.7	14.5	13.6	13.9
Aldehydes	1	1.7	1.5	1.4	1.1
Ketones	19.2	21.2	20.8	19.4	19.4
Esters	7.6	7.6	7.8	8.7	7.8
Furans	11.4	12.5	10.9	10.4	10.3
Dioxolanes	2.6	2.6	2.4	2.5	2.4
Hydrocarbons	0	0	0.4	0.9	1.1
Sugars	15.3	12.6	13.6	14	14.7
N-containing species	7.7	7.4	7	8.3	7.8
Others	2.1	1.9	1.9	2	1.8

## Data Availability

Data available on reasonable request from the corresponding author.

## Supplementary Materials

The following supporting information can be downloaded at: https://www.mdpi.com/article/10.3390/polym14225035/s1, Figure S1. Denim jeans waste used as the feedstock in this study; Figure S2. Scheme of the pyrolyser used for the pyrolysis of denim jeans waste; Table S1. Column information, and analytical conditions for the micro GC; Table S2. Column information, and analytical conditions for the GC/MS.
