# Supplementary material for: Pyrolysis of Denim Jeans Waste: Pyrolytic Product Modification by the Addition of Sodium Carbonate
Source: Polymers (Basel). 2022 Nov 21;14(22):5035. doi: 10.3390/polym14225035 (PMC9698909; doi:10.3390/polym14225035)
Supplement: Supplementary file 1 [file polymers-14-05035-s001.zip › polymers-2031836-supplementary.pdf]

polymers

MDPI

Supplementary Materials

Pyrolysis of Denim Jeans Waste: Pyrolytic Product Modification by the Addition of Sodium Carbonate

Junghee Joo [1], Heeyoung Choi [1], Kun-Yi Andrew Lin [2] and Jechan Lee [1,3,*]

[1] Department of Global Smart City, Sungkyunkwan University, Suwon 16419, Republic of Korea
[2] Department of Environmental Engineering & Innovation and Development Center of Sustainable Agriculture, National Chung Hsing University, Taichung, Taiwan
[3] School of Civil, Architectural Engineering, and Landscape Architecture, Sungkyunkwan University, Suwon 16419, Republic of Korea
* Correspondence: jechanlee@skku.edu

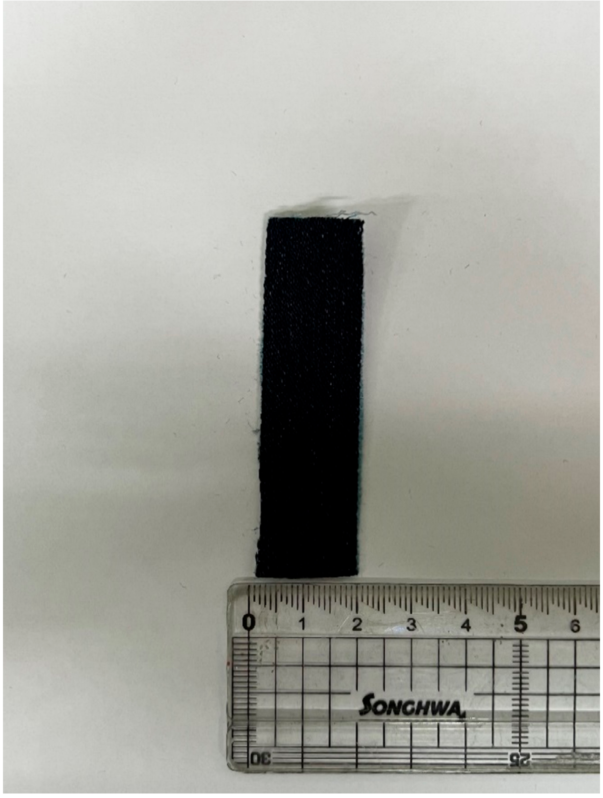

Figure S1. Denim jeans waste used as the feedstock in this study.

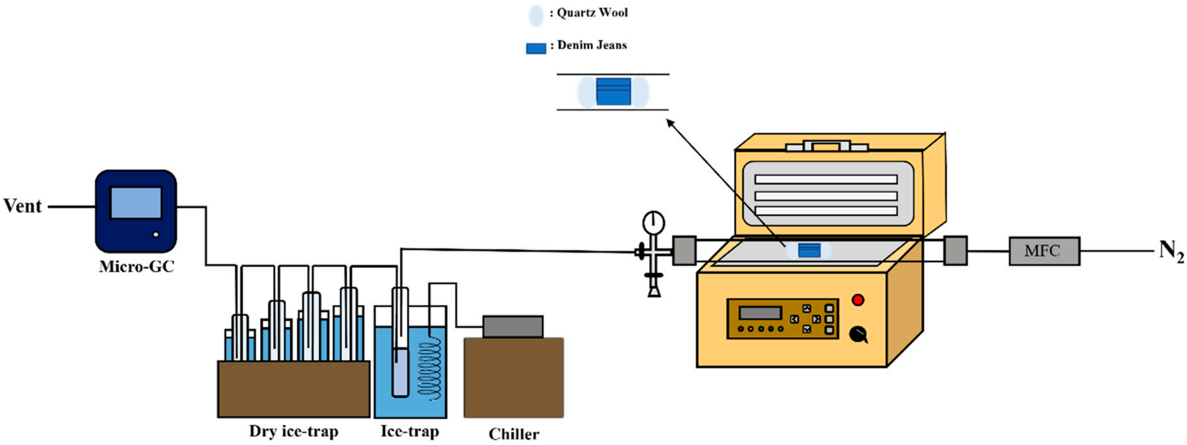

Figure S2. Scheme of the pyrolyser used for the pyrolysis of denim jeans waste.

Table S1. Column information, and analytical conditions for the micro GC.

**Model**		**INFICON Fusion Gas Analyzer**	
**Conditions**		**Module A**	**Module B**
**Column**		**Rt-Molsieve 5A**	**Rt-Q-Bond**
**Sample Pump setting**	Sample pump mode	Continuous	Continuous
	Sample pump time	15 s	15 s
**Column setting**	Carrier gas	Argon (≥99.999%)	Helium (≥ 99.999%)
	Column pressure	20 psi	17 psi
	Initial temperature	50 °C (40 s)	50 °C (30 s)
	Ramping time	50 s	60 s
	Final temperature	100 °C (40 s)	110 °C (40 s)
	Total analysis time	130 s	130 s
**Injector setting**	Injector temperature	90 °C	90 °C
	Inject time	30 ms	30 ms
**TCD setting**	TCD temperature	70 °C	70 °C
	Data rate	50 Hz	50 Hz

Table S2. Column information, and analytical conditions for the GC/MS.

**Model**	GC: Agilent 8890; MS: Agilent 5977B	
**Column**	HP-5MS Ultra Inlet column (0.25 mm × 0.25 um × 30 m)	
**Oven setting**	Initial temperature	30 °C (10 min)
	Ramping	5 °C min^{-1}
	Final temperature	300 °C (10 min)
	Total analysis time	74 min
**Column setting**	Column flow	1 mL min^{-1}
	Carrier gas	Helium (≥99.999%)
	Carrier gas flow	3 mL min^{-1}
**Injector setting**	Injection mode	Splitless
	Injection volume	1 µL
	Injection temperature	280 °C
**MS setting**	m/z range	35~550 amu
	Aux temperature	300 °C
